# Successful Management of Pancreatic Ascites with both Conservative Management and Pancreatic Duct Stenting

**DOI:** 10.4021/gr2009.08.1306

**Published:** 2009-07-20

**Authors:** Kalyan Kanneganti, Sirisha Srikakarlapudi, Bijay Acharya, Venkatram Sindhaghatta, Sridhar Chilimuri

**Affiliations:** a Division of Gastroenterology, Bronx-Lebanon Hospital Center affiliated to the Albert Einstein College of Medicine, Bronx, NY, USA; b Department of Medicine, Bronx-Lebanon Hospital Center affiliated to the Albert Einstein College of Medicine, Bronx, NY, USA; c Division of Pulmonary Medicine, Bronx-Lebanon Hospital Center affiliated to the Albert Einstein College of Medicine, Bronx, NY, USA

**Keywords:** Pancreatic ascites, Stent, Conservative management

## Abstract

Pancreatic ascites is a rare complication and should be suspected in patients with chronic alcoholism and pancreatitis presenting with ascites. The etiology is likely from a pancreatic pseudocyst leakage or due to ductal disruption. Treatment is controversial but includes conservative medical therapy or endoscopic transpapillary pancreatic duct stenting or surgery. We present a case of pancreatic ascites in a patient with alcohol use and chronic pancreatitis. Patient received conservative therapy including octreotide. An endoscopic retrograde cholangiopancreatography was performed, which confirmed a pancreatic duct dehiscence with extravasation of the injected contrast. This was treated with placement of a stent. Patient improved clinically and symptomatically. This case report augments the existing data from two prior reported case series, and this modality of management should be actively pursued in such cases.

## Introduction

Pancreatic ascites is a rare complication and should be suspected in patients with chronic alcoholism and pancreatitis presenting with ascites. The etiology is likely from a pancreatic pseudocyst leakage or due to ductal disruption. Medical treatment includes holding oral feedings, total parenteral nutrition, paracentesis and administering octreotide. Interventional therapy includes endoscopic transpapillary pancreatic duct stenting or surgery which includes cystgastrostomy, cystenterostomy or partial pancreatic resection. We present a case of pancreatic ascites in a patient with alcohol use and chronic pancreatitis. This was successfully managed with a combination of medical and interventional therapy including endoscopic pancreatic duct stenting.

## Case report

**Figure 1:**
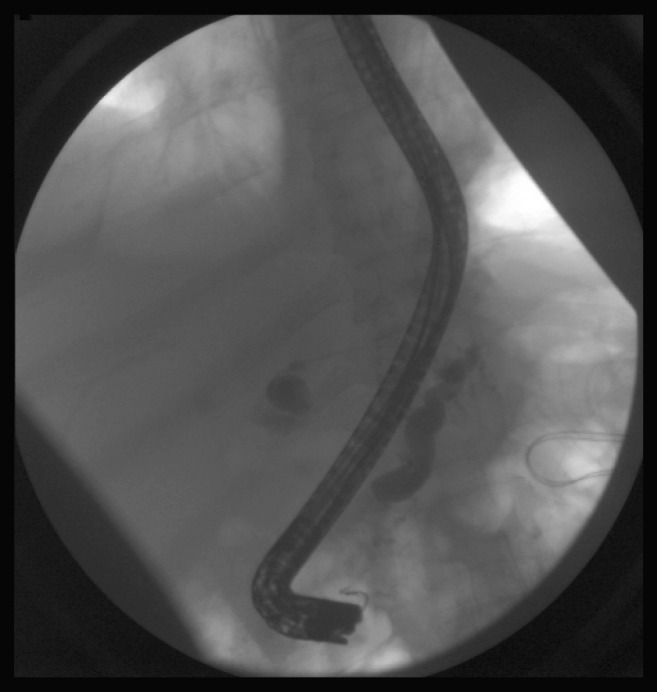
Pancreatic duct dehiscence with extravasation of contrast.

A 45 years old man presented with one month history of generalized abdominal pain and increasing abdominal distension. Three days prior to admission the pain was worsening in severity with associated nausea and constipation. The patient denied any fever, chills or gastrointestinal bleeding. His prior medical history included chronic pancreatitis, liver cirrhosis, polysubstance use including alcohol and multiple episodes of seizures. The patient had many prior admissions in our hospital for chronic pancreatitis and was diagnosed with a pancreatic pseudocyst and bacterial peritonitis in one prior admission. The patient continued to consume alcohol until the current admission despite alcohol cessation counseling multiple times on prior admissions. Physical examination was remarkable for marked abdominal distension with mild generalized tenderness and shifting dullness. Laboratory findings revealed elevated white blood cell count, abnormal liver function tests and elevated amylase and lipase levels. Contrast CT scan of abdomen revealed acute on chronic pancreatitis with extra-hepatic biliary duct and main pancreatic ductal dilatation. The previously noted pseudocyst was resolved with interval development of large amount of ascites with partial small bowel obstruction. A diagnostic and therapeutic paracentesis was performed. Ascitic fluid analysis revealed a white blood cell count of 2470 cells/mm^3^ with 98% granulocytes, total protein was > 3g/dL. Ascitic fluid amylase was > 20,000 IU/L which increased to 35,000 IU/L on a subsequent paracentesis. Ascitic fluid culture grew *Streptococcus viridans* which was treated with antibiotics for two weeks. Patient was managed conservatively including holding oral feedings and octreotide infusion but continued to have worsening ascites and required repeated therapeutic paracentesis. A diagnosis of pancreatic ascites with possible pancreatic duct dehiscence was made. An endoscopic retrograde cholangiopancreatography (ERCP) was performed, which confirmed a duct dehiscence with extravasation of the injected contrast (Fig. 1) and was treated with placement of a stent. Patient improved clinically and symptomatically with a repeat paracentesis showing decreased amylase levels. On a follow-up clinic visit, patient was noted to have marked improvement in abdominal distension and discomfort.

## Discussion

Ascites is commonly seen in patients with alcoholic liver disease and is usually a consequence of portal hypertension. Pancreatic ascites is a rare diagnostic possibility in patients with chronic alcoholism and pancreatic disease. Pancreatic ascites refers to the accumulation of peritoneal fluid in the presence of pancreatic disease and is usually secondary to pancreatic pseudocysts or pancreatic duct dehiscence. This was first reported in the literature in 1953 in two cases of chronic pancreatitis associated with ascites [1]. Pancreatic ascites is an exudative ascites characterized by high amylase concentration in ascitic fluid (usually over 1000 IU/L) and protein concentration over 3 g/dl [2, 3] that differentiates it from ascites secondary to cirrhosis, tuberculosis or carcinomatosis. Rarely, the origin is indeterminate in 10% of cases [4-6]. In addition to ascites, pleural effusions were seen. Pancreatic ascites is infrequent and is reported in 3.5% of patients with chronic pancreatitis and 6 – 14% of patients with pseudocysts [7, 8].

Causes for pancreatic ascites include chronic pancreatitis, pancreatic trauma and cystic duplications of biliopancreatic ducts, ampullary stenosis, or ductal lithiasis [3]. Pancreatic ascites is more prevalent in men (male:female ratio 2:1) and between 20 - 50 years of age [3]. In children, it is uncommon and is usually caused by abdominal trauma or congenital obstruction of the pancreatic duct [9]. Acute pancreatitis can be complicated with transient ascites rich in pancreatic enzymes; however, this does not usually cause pancreatic ascites.

The clinical presentation includes mild abdominal pain, weight loss and progressive ascites. Severe pain is unusual. Pancreatic ascites should be considered in the differential in patients with chronic ascites with history of alcoholism, chronic pancreatitis or abdominal trauma [10].

Therapy for pancreatic ascites is controversial [11]. There are no randomized control studies regarding therapy due to the rarity of the condition. Though no consensus is established on the management, Chebli et al [12] propose that the approach depends on the endoscopic retrograde pancreatography defined pancreatic ductal anatomy.

Conservative therapy includes the use of somastatin analogues [13, 14] to decrease pancreatic secretion, keeping the patient Nil Per Oral (NPO) with parenteral nutrition and repeated ascitic fluid drainage. Initial continuous octreotide infusions followed by subcutaneous injections are suggested. Long acting octreotide designed for once a month intra-muscular administration is an option. The total duration of therapy is unknown and a trial of 2 - 4 weeks is suggested with a consideration for interventional therapy if no resolution.

The interventional therapy is either endoscopic or surgical. ERCP is a valuable tool in the evaluation of patients with pancreatic ascites. It delineates the anatomy, shows communication to the pseudocyst and reveals disruption of the pancreatic duct. Patients undergoing ERCP usually do better than those without ERCP [15]. Increased pressure within the pseudocysts or the pancreatic duct results in disruption of pancreatic duct, and ERCP reduces intraductal pressure by sphincterotomy or by insertion of a transpapillary stent [16]. In addition, pre-operative ERCP is recommended before surgery to decide on the approach based on the location of the leak.

Surgical therapy for pancreatic ascites is usually recommended early when the etiology is due to trauma. Surgical therapy is also recommended when there is no response to conservative therapy for 3 - 4 weeks. Pseudocysts are usually treated by distal pancreatectomy when the leak is in the pancreatic tail or drained by cystogastrostomy, cystojejunostomy, or cystoduodenostomy. Internal drainage is preferred to external drainage. Fistulas in the pancreatic duct are usually drained to a Roux-en-Y jejunal loop. Recurrence rates from 50 - 64% have been reported in patients undergoing surgical intervention without ERCP [15, 17]. Mortality rates have been reported to be similar with surgical and medical therapies (15 - 25%) [18].

In this case report we successfully managed a patient with pancreatic ascites with a combination of conservative and interventional therapy including an ERCP with pancreatic ductal stenting. Our case report augments the existing data from two prior reported case series [19, 20], and this modality of management should be actively pursued in such cases. However, a larger number of patients and randomized controlled trials are necessary to confirm this.
